# Cyclic dinucleotides (CDNs) anti-tumors response by activating DC and NK cell crosstalk

**DOI:** 10.1186/2051-1426-2-S3-P169

**Published:** 2014-11-06

**Authors:** Juan Fu, Drew Pardoll, Young J Kim

**Affiliations:** 1 Department of Otolaryngology - Head & Neck Surgery, Johns Hopkins University, School of Medicine, Baltimore, MD, USA; 2 Department of Oncology and the Sidney Kimmel Comprehensive Cancer Center, Johns Hopkins University, School of Medicine, Baltimore, MD, USA

## 

Intracellular bacterial, *Listeria monocytogenes *generates cyclic diadenosine monophosphate (c-di-AMP) can active interferon regulatory factor3 (IRF3) and nuclear factor kappa-light-chain-enhancer (NF-κB) and induces B cell and macrophage secretion of IFN-β [1]. Cyclic diguanylic acid (c-di-GMP) also acts as an important signaling molecule in a variety of bacterial species infection functions. IFN-β actives NK cells through Tyk_2_-STAT1 signal pathway. Our studied showed CDNs anti-tumor effective dependent IFNα/β receptors (IFNAR1/IFNAR2) on the cell plasma membrane. Some study showed c-di-GMP significantly inhibited the proliferation of human colon cancer cells in vitro [2]. Cyclic dinucleotides (CDNs, c-di-AMP and c-di-GMP) are sensed by STING (stimulator of interferon genes). But CDNs were developed for prevent and therapeutic cancers, it was a novel method. We combined GM-CSF-producing tumor vaccine and TLR agonists enhanced systemic anti-tumor immunity. Our studied showed the regimen significantly inhibition mice tumors growth in B16 melanoma and colon cancer in vivo.
